# Tailoring the Electronic and Structural Properties of Lead-Free A_2_ZrX_6_ “Defect” Perovskites: A DFT Study on A-Site Cation and Halogen Substitutions

**DOI:** 10.3390/ma18173976

**Published:** 2025-08-25

**Authors:** Christina Kolokytha, Demeter Tzeli, Nektarios N. Lathiotakis

**Affiliations:** 1 Theoretical and Physical Chemistry Institute, National Hellenic Research Foundation, 48 Vassileos Constantinou Ave., GR-11635 Athens, Greece; kolokythac28@chem.uoa.gr; 2 Laboratory of Physical Chemistry, Department of Chemistry, National and Kapodistrian University of Athens, GR-15784 Zografou, Greece

**Keywords:** hybrid organic–inorganic perovskites, materials for energy applications, optoelectronic properties, hybrid DFT calculations

## Abstract

Lead-free A_2_ZrX_6_ “defect” perovskites hold significant potential for many optoelectronic applications due to their stability and tunable properties. Extending a previous work, we present a first-principles density functional theory (DFT) study, utilizing PBE and HSE06 functionals, to systematically investigate the impact of A-site cation and X-site halogen substitutions on the structural and electronic properties of these materials. We varied the A-site cation, considering ammonium, methylammonium, dimethylammonium, trimethylammonium, and phosphonium, and the X-site halogen, trying Cl, Br, and I. Our calculations reveal that both these substitutions significantly affect the band gap and the lattice parameters. Increasing A-site cation size generally enlarges the unit cell, while halogen electronegativity directly correlates with the band gap, yielding the lowest values for iodine-containing systems. We predict a broad range of band gaps (from ~4.79 eV for (PH_4_)_2_ZrCl_6_ down to ~2.11 eV for MA_2_ZrI_6_ using HSE06). The (PH_4_)_2_ZrX_6_ compounds maintain cubic crystal symmetry, unlike the triclinic of the ammonium-derived systems. Finally, our calculations show that the MA cation yields the smallest band gap among the ones studied, a result that is attributed to its size and the charges of the hydrogen atoms attached to nitrogen. Thus, our findings offer crucial theoretical insights into A_2_ZrX_6_ structure–property relationships, demonstrating how A-site cation and halogen tuning enables control over electronic and structural characteristics, thus guiding future experimental efforts for tailored lead-free perovskite design.

## 1. Introduction

Metal-halide perovskites remain a focal point of intense research, due to their great potential in various applications, including photovoltaics [1,2,3], light-emitting diodes (LEDs) [4,5,6], photodetectors (PDs) [7], laser crystals [4], transistors [8], memories [9], superconductors [10],photocatalysts [11], and other optoelectronic devices [12,13]. Typical metal-halide perovskites are of the form ABX_3_ where the A-site is occupied by inorganic (e.g., cesium) or organic (e.g., methylammonium (MA), formamidinium (FA)) cations, the B-site typically houses a divalent metal (e.g., Pb^2+^, Sn^2+^, Ge^2+^), and the X-site hosts a halogen anion (Cl, Br, I).

In photovoltaics, metal-halide perovskites have rapidly achieved Power Conversion Efficiency (PCE) that exceeds 25% in single-junction laboratory devices, and even higher in tandem configurations [14]. In organic–inorganic hybrid perovskites, high efficiency is achieved in parallel with low fabrication cost; however, these systems are facing challenges with operational stability [15]. In light-emitting diodes (LEDs), they have also achieved external quantum efficiency (EQE) exceeding 20% in the green and red spectrum, approaching the efficiencies of established OLEDs [16,17]. As photo-detectors [7,17], they have the potential to replace traditional photo-detection materials such as silicon, III–V, or II–VI semiconductors. The potential of metal-halide perovskites in these applications is based on their compelling features, including low fabrication cost through solution-based manufacturing, inherent flexibility, and tunable optoelectronic properties. Among them, lead-halide perovskites have demonstrated exceptional performance. However, the adoption of technologies based on these materials is challenged by concerns regarding the toxicity of lead and the inherent instability of lead-halide perovskites when exposed to environmental stressors like moisture and oxygen [18,19]. To address these challenges, it is crucial to develop environmentally stable, robust, and non-toxic perovskite materials capable of matching or surpassing the performance of their lead-containing counterparts [20,21].

A promising direction in lead-free perovskite research concerns the so-called double perovskites materials, with the general formula A_2_BB^’^O_6_, where A-site cations occupy interstitial spaces within a three-dimensional network of alternating BO_6_ and B^’^O_6_ octahedra [22]. Double perovskites have demonstrated strong and unusual magnetic interactions, enhanced stability, and are considered as viable options to replace lead halide perovskites [22,23,24]. Closely related structures are the “defect perovskites”, for which the B^’^ site is vacant and a tetravalent metal cation (Sn^+4^, Ti^+4^, Zr^+4^) occupies the B position to maintain charge neutrality [13,25,26]. They are also known as vacancy-ordered double perovskites, with the general formula A_2_BX_6_, and have also received significant attention [13,25,26]. Unlike their ABX_3_ counterparts, they feature isolated [BX_6_] octahedra, which often translates to enhanced structural stability, while due to the “vacancy” sites a large variety of A cations can be accommodated. Within this class, metal halide perovskites based on non-toxic and abundant Zirconium (A_2_ZrX_6_) represent an attractive proposition for optoelectronic applications [5,27,28,29,30].

Zhu et al. [31] developed Zr-based perovskites achieving high photoluminescence quantum yields (PLQYs). However, despite their promise, challenges remain in optimizing synthesis and addressing issues like color purity and long-term stability under light exposure. The electronic properties of Cs_2_BX_6_, with B = Sn, Te, Zr, were studied theoretically [26] and it was found that they can be tailored for hole and electron transport. Cs_2_ZrX_6_ (X = Cl, Br) perovskite derivatives were synthesized and studied, theoretically, by Abfalterer et al. [27], showing good agreement between theory and experiment. For these compounds, indirect band gaps of the order of 4–5 eV were found. Dai et al. [32] reported the synthesis of ((CH_3_)_4_N)_2_ZrCl_6_ and demonstrated that it exhibits excitation-dependent fluorescence across the visible region. Lin et al. [33] focused on the optical property regulation of 0D A_2_ZrCl_6_ through non-protonated cation substitution (A = [(CH_3_)_3_SO]^+^ and [(CH_3_)_4_N]^+^) combined with different guest ions (i.e., Sb^3+^, Bi^3+^, and Mn^2+^), providing insights into compositional engineering. Another zirconium-containing perovskite, ((CH_3_)_2_S)_2_ZrCl_6_, was recently created, by Tagiara et al. [34] and exhibited broad photoluminescence. Finally, a newly developed zero-dimensional organic–inorganic hybrid perovskite [5], ((C_2_H_5_)_4_N)_2_ZrCl_6_, was found to exhibit multiple emissions in red, green, and blue, attributed to different mechanisms, like self-trapped excitons, Zr(IV) *d*–*d* transitions, and thermally activated delayed fluorescence (TADF), and boasted a high photoluminescence quantum yield.

Inspired by recent experimental results [34], in a previous work [35], we studied theoretically “defect” perovskites, with the chemical formula A_2_ZrX_6_, where the A-site cation is either methylammonium, formamidinium, or trimethyl-sulfonium, and the X-site anions are halogen, X = Cl, Br, and I, using hybrid density functional theory (DFT). The target of our study was the exploration of the effect of the A-site cation as well as the X-site anion on the electronic properties. We found that all compounds exhibit wide band gaps ranging from 5.22 eV down to 2.11 eV. One main point was that both the A-site cation as well as the X-site anion can be used to tailor the electronic properties [35]. Among the studied systems, the particular one that combines methylammonium cation and iodine anion was found to yield the lowest band gap.

Motivated by the findings of our previous work [35], especially the pronounced influence of ion substitution on band gaps, the present study extends the theoretical investigation of A-site cation substitutions in A_2_ZrX_6_ perovskites, employing DFT methods. Prompted by the approximately 2 eV band gap calculated for methylammonium (MA), CH_3_NH_3_^+^, in the A-site, we expand our focus to include other ammonium-derived cations, namely ammonium, NH_4_^+^, dimethylammonium (DMA), (CH_3_)_2_NH_2_^+^, and trimethylammonium (TMA), (CH_3_)_3_NH^+^, as well as the related phosphonium cation, PH_4_^+^. For completeness, in order to have all ammonium-derived cations up to TMA, our results, already presented in Ref. [35] concerning MA, are also included. These cations are combined with halide anions X = Cl, Br, and I. For these systems, we predict the structural properties, as well as basic electronic properties (densities of states, band gaps). Although there are several reports of utilizing such cations in metal-halide perovskites [36], there is a scarcity of results concerning these cations in Zr-based systems. It is our ambition to motivate such experimental and theoretical studies through this work, by demonstrating the tunability of the electronic properties of these systems. Furthermore, our study aims to categorize the considered perovskite materials based on their bandgap to determine their suitability for specific applications. Materials with bandgaps in the range 1.5–2.8 eV are suitable for solar cells, LEDs, and thin-film transistors. Specifically, a 2.5 eV bandgap is optimal for blue light absorption, while a 1.9 eV band gap targets red light. On the other hand, a bandgap of 1.5 eV enables broad absorption across visible and near-infrared regions and can be versatile for energy and optoelectronic applications.

## 2. Computational Methodology

We performed DFT calculations using the Vienna Ab initio Simulation Package (VASP) plane-wave code [37,38], version 6.1, employing the projected augmented wave (PAW) formalism [39]. First, every perovskite structure was fully energetically optimized, allowing the relaxation of every atomic position and the unit cell shape and volume, using the Perdew–Burke–Ernzerhof (PBE) generalized gradient approximation (GGA). For the electronic properties of the optimized structures, apart from GGA-PBE functional, which systematically underestimates band gaps, we employed the Heyd–Scuseria–Ernzehof (HSE06) screened hybrid functional [40]. It has been established that hybrid functionals predict accurately optoelectronic properties [41,42]. In our previous work [35], we demonstrated that the strategy of using the PBE functional for the energetic optimization of the structures and HSE06 for the electronic properties of these systems is accurate compared to experimental results. The convergence criterion for the electronic self-consistency cycle was set at 10^−5^ eV, while the ionic relaxation was terminated when the total energy change was less than 10^−4^ eV. Furthermore, we used the value of 400 eV for the maximum energy cutoff and 2 × 2 × 2 reciprocal space sampling, and these choices were also validated in our previous work.

## 3. Results and Discussion

### 3.1. (NH_4_)_2_ZrX_6_, X: Cl, Br, I

**Figure 1:**
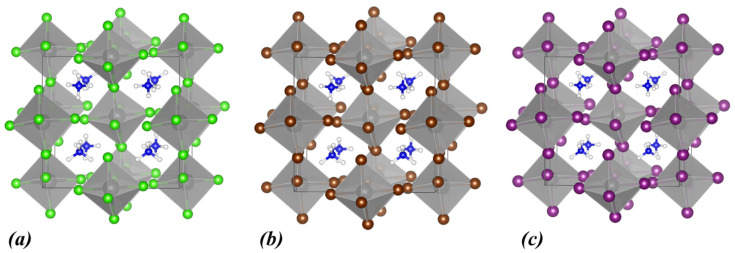
Calculated crystal structures of (**a**) (NH_4_)_2_ZrCl_6_, (**b**) (NH_4_)_2_ZrBr_6_, and (**c**) (NH_4_)_2_ZrI_6_. In the polyhedral structures, Zr is depicted as grey spheres, and the halogen atoms are shown as green (Cl), brown (Br), and purple (I) spheres. Within the ammonium cation, N atoms are shown as blue and H atoms as white spheres.

**Figure 2:**
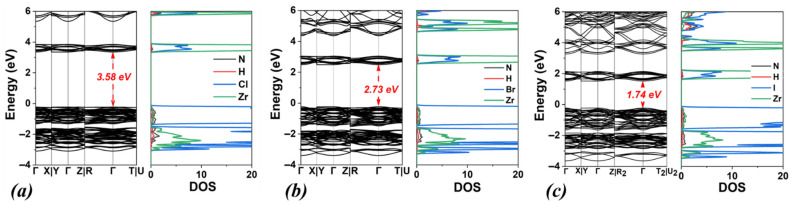

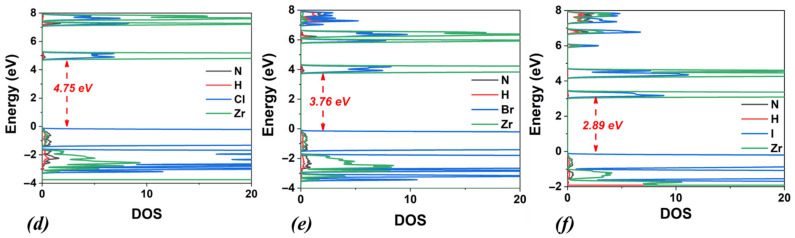
Calculated band structures and densities of states (DOSs) using the PBE functional for (**a**) (NH_4_)_2_ZrCl_6_, (**b**) (NH_4_)_2_ZrBr_6_ and (**c**) (NH_4_)_2_ZrI_6_. Corresponding DOSs calculated using the HSE06 functional are also shown for (**d**) (NH_4_)_2_ZrCl_6_, (**e**) (NH_4_)_2_ZrBr_6_ and (**f**) (NH_4_)_2_ZrI_6_.

Initially, our computational study focused on the (NH_4_)_2_ZrX_6_ system, i.e., with ammonium as the A-site cation and X = Cl, Br, and I. The energy optimized structures are shown in Figure 1, while the obtained structural and electronic properties are included in Table 1. The calculated band structures and densities of states (DOSs) using the PBE functional for all halogen structures are shown in Figure 2a–c together with the DOS obtained with HSE06 (Figure 2d–f).

Regarding the structural properties of (NH_4_)_2_ZrX_6_, as seen in Table 1, the symmetry of the crystal structure is triclinic. Moreover, for chlorine the lattice constants are the smallest compared to the rest of the halogens. For Br, the lattice constants increase by approximately 0.5 Å. Finally, for I, an additional increase of about 0.8 Å, in the lattice constants is predicted. Overall, we can conclude that as the ionic radius of the halogen atoms increases (R_I_ > R_Br_ > R_Cl_), the lattice constants increase accordingly.

Concerning the electronic properties, the bandgap energy values calculated by the PBE functional are underestimated compared to those obtained by the HSE06, as expected (see Table 1) by approximately 1–1.2 eV. Moreover, as the electronegativity of the halogen decreases (Cl > Br > I), the bandgap energy shows a reduction by approximately 1 eV, for both functionals. In particular, for the perovskite (NH_4_)_2_ZrCl_6_, the bandgap energies are 3.58 eV (PBE) and 4.75 eV (HSE06). When Cl anions are replaced by Br, the bandgap energies decrease by 0.8 eV (to 2.73 eV) using PBE and by 1.0 eV (to 3.76 eV) using HSE06. Finally, for the perovskite (NH_4_)_2_ZrI_6_, the calculated values of the bandgap energy are 1.74 eV (PBE) and 2.89 eV (HSE06), showing a decrease of 1.0 eV (PBE) and 0.9 eV (HSE06), compared to (NH_4_)_2_ZrBr_6_.

### 3.2. MA_2_ZrX_6_, X: Cl, Br, I

**Figure 3:**
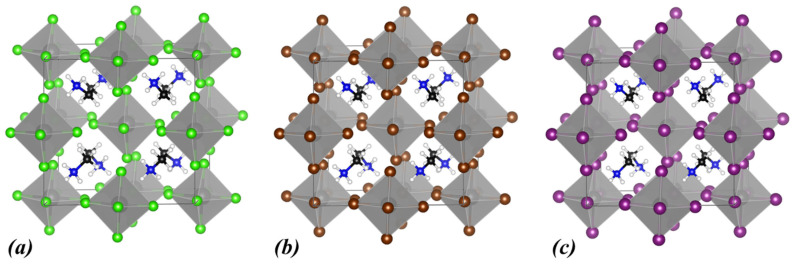
Calculated crystal structures of (**a**) MA_2_ZrCl_6_, (**b**) MA_2_ZrBr_6_, and (**c**) MA_2_ZrI_6_. In the polyhedral structures, Zr is depicted as grey spheres, and the halogen atoms are shown as green (Cl), brown (Br), and purple (I) spheres. Within MA^+^, N atoms are shown as blue, C atoms as black, and H atoms as white spheres.

**Figure 4:**
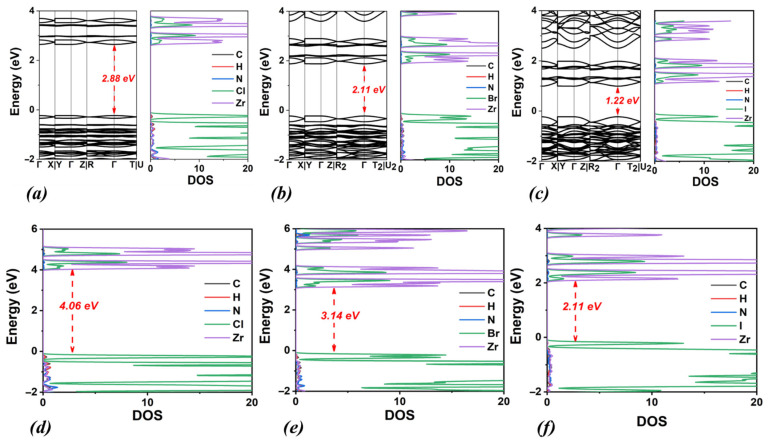
Calculated band structures and densities of states (DOSs) using the PBE functional for (**a**) MA_2_ZrCl_6_, (**b**) MA_2_ZrBr_6_ and (**c**) MA_2_ZrI_6_. Corresponding DOSs calculated using the HSE06 functional are also shown for (**d**) MA_2_ZrCl_6_, (**e**) MA_2_ZrBr_6_, and (**f**) MA_2_ZrI_6_.

Our next focus is the MA cation at the A-site, formed by replacing a hydrogen atom of the NH_4_ ion with a methyl group. The energetically optimal structures of MA_2_ZrCl_6_ are shown in Figure 3, while the structural and electronic properties are included in Table 2 and Figure 4. In the case of MA_2_ZrCl_6_, the calculated values of the bandgap energy are 2.88 eV (PBE) and 4.06 eV (HSE06) which are lower than those of (NH_4_)_2_ZrCl_6_ by 1.3 eV and 0.7 eV, respectively. Furthermore, for the bandgap energy of MA_2_ZrBr_6,_ we calculated the values 2.11 eV (PBE) and 3.14 eV (HSE06), which are lower than those of (NH_4_)_2_ZrBr_6_ by 0.6 eV, for both approximations. Finally, for the bandgap energy of MA_2_ZrI_6,_ we found the values 1.22 eV (PBE) and 2.11 eV (HSE06) which are lower by 0.6 eV and 0.7 eV, respectively, compared to (NH_4_)_2_ZrI_6_. In conclusion, by replacing NH_4_^+^ with MA^+^, the bandgap energies drop substantially for all the halogen options.

In addition, replacing NH_4_^+^ with MA^+^ at the A-site, significantly affects the structural properties. For all studied halogens (Cl, Br, and I), the lattice constants of the MA_2_ZrX_6_ structures are consistently larger than their (NH_4_)_2_ZrX_6_ counterparts (see Table 1 and Table 2). Specifically, the lattice constants for MA_2_ZrCl_6_ increase by 0.7–0.9 Å compared to (NH_4_)_2_ZrCl_6_. Similarly, MA_2_ZrBr_6_ shows an increase of 0.6–1.0 Å relative to (NH_4_)_2_ZrBr_6_, and MA_2_ZrI_6_ exhibits a comparable increase of 0.6–1.0 Å over (NH_4_)_2_ZrI_6_. This expansion of the unit cell can be attributed to the larger molecular weight of the MA^+^ (32 g/mol) compared to NH_4_^+^ (18 g/mol). Despite the increase in unit cell size, the crystal symmetry remains triclinic.

### 3.3. DMA_2_ZrX_6_, X: Cl, Br, I

**Figure 5:**
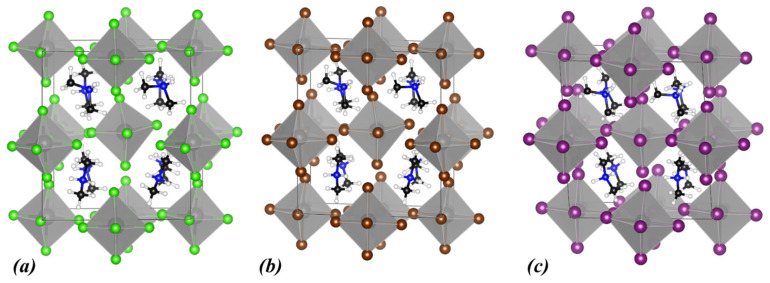
Calculated crystal structures of (**a**) DMA_2_ZrCl_6_, (**b**) DMA_2_ZrBr_6_, and (**c**) DMA_2_ZrI_6_. In the polyhedral structures, Zr is depicted as grey spheres, and the halogen atoms are shown as green (Cl), brown (Br), and purple (I) spheres. Within DMA^+^, N atoms are shown as blue, C atoms as black, and H atoms as white spheres.

**Figure 6:**
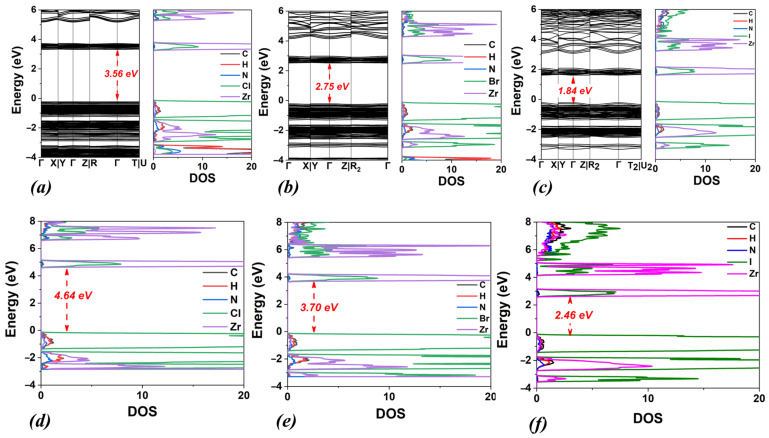
Calculated band structures and densities of states (DOSs) using the PBE functional for (**a**) DMA_2_ZrCl_6_, (**b**) DMA_2_ZrBr_6_, and (**c**) DMA_2_ZrI_6_. Corresponding DOS calculated using the HSE06 functional are also shown for (**d**) DMA_2_ZrCl_6_, (**e**) DMA_2_ZrBr_6_ and (**f**) DMA_2_ZrI_6_.

Next, we present results for DMA cation at the A-site, formed by replacing one more hydrogen in the MA^+^ with a methyl group. In Figure 5, the optimal structures for the three halogens are shown, and in Table 3 as well as in Figure 6, we present the structural characteristics and electronic properties.

Regarding the electronic properties, for DMA_2_ZrCl_6_, the bandgap energies are 3.56 eV (PBE) and 4.64 eV (HSE06). Compared to (NH_4_)_2_ZrCl_6_ (Figure 2, Table 1), DMA_2_ZrCl_6_ shows a slight decrease in bandgap energy, ranging from 0.02 to 0.11 eV. Instead, relative to MA_2_ZrCl_6_ (Table 2, Figure 4), the band gap of DMA_2_ZrCl_6_ increases by approximately 0.7 eV (PBE) and 0.6 eV (HSE06). For DMA_2_ZrBr_6_, the band gap values are 2.75 eV (PBE) and 3.70 eV (HSE06). Compared to (NH_4_)_2_ZrBr_6_ (see Table 1), the bandgap slightly increases by 0.02 eV for the PBE functional and decreases marginally, by 0.06 eV, for HSE06. On the contrary, relative to MA_2_ZrBr_6_ (Table 2), DMA_2_ZrBr_6_ shows a sizable increase in band gap by 0.64 eV (PBE) and 0.56 eV (HSE06). Finally, for DMA_2_ZrI_6_, the calculated bandgap values are 1.84 eV (PBE) and 2.46 eV (HSE06). These values are larger than those for MA_2_ZrI_6_ by 0.6 eV and 0.35 eV for PBE and HSE06, respectively. Following the bandgap trend with halogen substitutions, the DMA_2_ZrI_6_ bandgap values are substantially smaller than DMA_2_ZrBr_6_ and DMA_2_ZrCl_6_, as seen in Table 3. Overall, across all halogens, the band gap values of DMA_2_ZrX_6_ systems generally lie between those of the (NH_4_)_2_ZrX_6_ and MA_2_ZrX_6_ (for HSE06).

Regarding the structural properties of the DMA_2_ZrX_6_ compounds (Table 3), the crystal symmetry consistently remains triclinic. In the case of DMA_2_ZrCl_6_, the lattice constants are 11.21 Å, 12.68 Å, 11.41 Å for *a*, *b* and *c*, respectively, and they are higher than those of MA_2_ZrCl_6_ by ~2 Å and (NH_4_)_2_ZrCl_6_ by ~2.5 Å. In the case of Br in the X-site, the lattice constants are 11.55 Å, 13.29 Å, 11.64 Å, i.e., they are 0.2–0.8 Å larger than those of Cl anion in the X-site. Compared to MA_2_ZrBr_6_, one lattice parameter (*b* in Table 3) is significantly enlarged, while the other two are only marginally smaller. DMA_2_ZrBr_6_ lattice parameters are also significantly larger than those of and (NH_4_)_2_ZrBr_6_ by 0.5 Å, 2.7 Å and 1.1 Å, for *a*, *b*, *c*, respectively. For DMA_2_ZrI_6_, the lattice constants values are 12.20 Å, 13.84 Å, 12.30 Å and they are significantly enlarged compared to DMA_2_ZrBr_6_ by 0.5–0.7 Å. They are also substantially larger than both (NH_4_)_2_ZrI_6_ and MA_2_ZrI_6_ by 0.8–2.0 Å, and 0.4–1.5 Å, respectively.

### 3.4. TMA_2_ZrX_6_, X: Cl, Br, I

**Figure 7:**
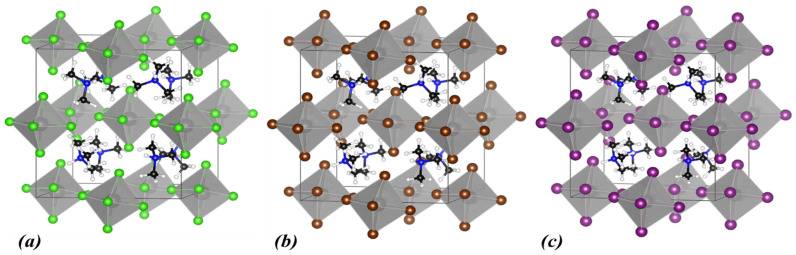
Calculated crystal structures of (**a**) TMA_2_ZrCl_6_, (**b**) TMA_2_ZrBr_6_, and (**c**) TMA_2_ZrI_6_. In the polyhedral structures, Zr is depicted as grey spheres, and the halogen atoms are shown as green (Cl), brown (Br), and purple (I) spheres. Within TMA^+^, N atoms are shown as blue, C atoms as black, and H atoms as white spheres.

**Figure 8:**
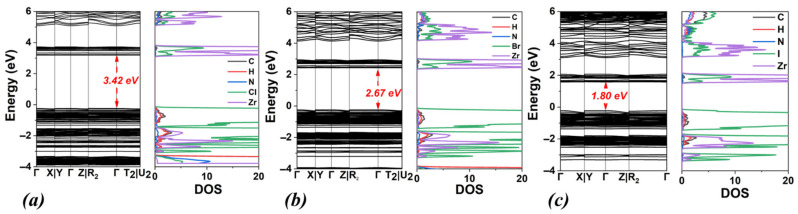

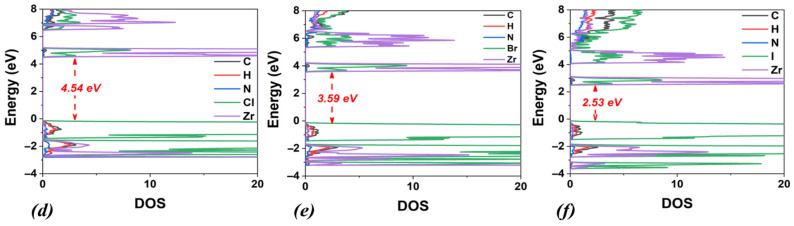
Calculated band structures and densities of states (DOSs) using the PBE functional for (**a**) TMA_2_ZrCl_6_, (**b**) TMA_2_ZrBr_6_, and (**c**) TMA_2_ZrI_6_. Corresponding DOSs calculated using the HSE06 functional are also shown for (**d**) TMA_2_ZrCl_6_, (**e**) TMA_2_ZrBr_6_ and (**f**) TMA_2_ZrI_6_.

Next, we investigated the TMA_2_ZrX_6_ series, where trimethylammonium (TMA^+^) occupies the A-site. The structures obtained for all halogens are shown in Figure 7. The electronic and structural properties for these structures are presented in Table 4 and Figure 8.

Figure 4 clearly illustrates that as the halogen electronegativity decreases, the band gap energy consistently decreases for both PBE and HSE06 functionals. Specifically, replacing chlorine (Cl) with bromine (Br) at the X-site causes the band gap energy to decrease by 0.8 eV (PBE) and 1.0 eV (HSE06). A similar reduction is observed when bromine (Br) is replaced by iodine (I), with the band gap energy decreasing by another 0.8 eV (PBE) and 1.0 eV (HSE06). Notably, the HSE06 functional consistently predicts a greater decrease in band gap energies compared to PBE.

For TMA_2_ZrCl_6_, the band gap energies are 3.42 eV (PBE) and 4.54 eV (HSE06). These values are slightly lower (by ~0.1 eV) than those of DMA_2_ZrCl_6_ (3.56 eV with PBE and 4.64 eV with HSE06). Similarly, TMA_2_ZrBr_6_ exhibits band gap energies of 2.67 eV (PBE) and 3.59 eV (HSE06), which are also lower by ~0.1 eV compared to DMA_2_ZrBr_6_ (2.75 eV with PBE and 3.70 eV with HSE06). Finally, for TMA_2_ZrI_6_, the calculated bandgap energy values are 1.80 eV (PBE) and 2.53 eV (HSE06).

Regarding the structural properties of the TMA_2_ZrX_6_ compounds (Table 4), the lattice constants are significantly influenced by the increasing molecular weight of the halogen at the X-site. Specifically, for TMA_2_ZrCl_6_, the lattice constants are *a* =11.64 Å, *b* =13.21 Å, and *c* =12.04 Å. When Cl is replaced by Br, all three lattice parameters increase by approximately 0.4 Å. Further substitution of Br with iodine I results in even larger lattice constants, *a* =12.67 Å, *b* =14.25 Å, and *c* =13.03 Å, representing an additional increase of 0.4–0.6 Å. Compared to DMA_2_ZrX_6_, the lattice parameters of TMA_2_ZrX_6_ are substantially enlarged: in the cases of Cl and Br anions, they are increased by 0.5 Å on the average, while, in the case of I, this expansion is more than 1.0 Å, for all parameters *a*, *b* and *c*.

### 3.5. (PH_4_)_2_ZrX_6_, X: Cl, Br, I

**Figure 9:**
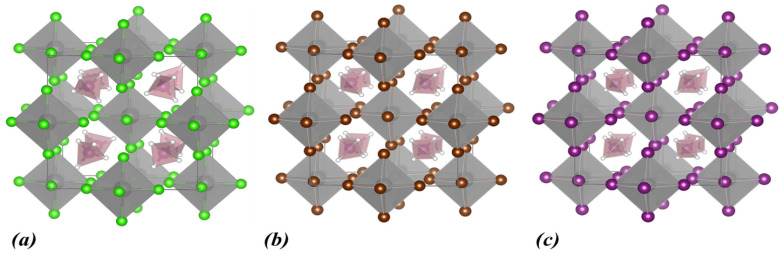
Calculated crystal structures of (**a**) (PH_4_)_2_ZrCl_6_, (**b**) (PH_4_)_2_ZrBr_6_, and (**c**) (PH_4_)_2_ZrI_6_. In the polyhedral structures, Zr is depicted as grey spheres, and the halogen atoms are shown as green (Cl), brown (Br), and purple (I) spheres. Within the (PH_4_)^+^, P atoms are shown as pink and H atoms as white spheres.

**Figure 10:**
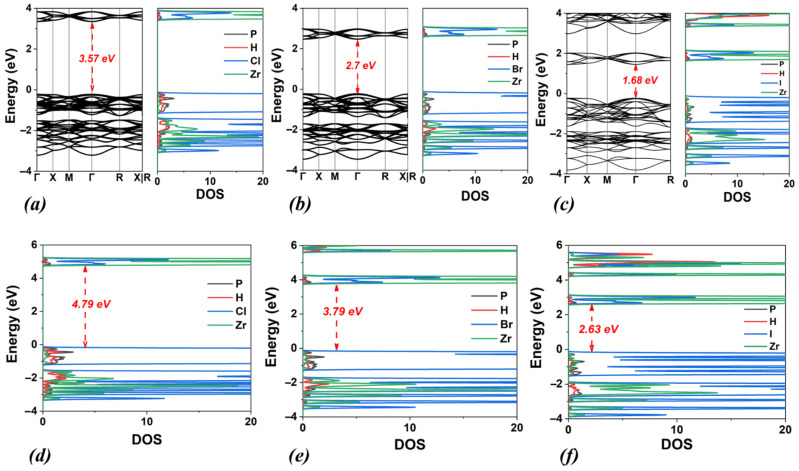
Calculated band structures and densities of states (DOSs) using the PBE functional for (**a**) (PH_4_)_2_ZrCl_6_, (**b**) (PH_4_)_2_ZrBr_6_, and (**c**) (PH_4_)_2_ZrI_6_. Corresponding DOSs calculated using the HSE06 functional are also shown for (**d**) (PH_4_)_2_ZrCl_6_, (**e**) (PH_4_)_2_ZrBr_6_, and (**f**) (PH_4_)_2_ZrI_6_.

The last crystal structure that we studied is (PH_4_)_2_ZrX_6_, where the phosphonium cation (PH_4_^+^) serves as the A-site cation. The structures we obtained from geometry optimization are shown in Figure 9, while our results are included in Table 5 and Figure 10.

Notably, (PH_4_)_2_ZrX_6_ compounds exhibit a unique structural feature: their crystal symmetry remains cubic regardless of the halogen (Table 5). This contrasts with the triclinic crystal symmetry observed for all the ammonium-derived A-site cations investigated (NH_4_^+^, MA^+^, DMA^+^, and TMA^+^).

Regarding their electronic properties (Figure 10 and Table 5), the calculated bandgap energy of (PH_4_)_2_ZrCl_6_ has values of 3.57 eV (PBE) and 4.79 eV (HSE06). These values are comparable to those of (NH_4_)_2_ZrCl_6_, DMA_2_ZrCl_6_, and TMA_2_ZrCl_6_ (Figure 2, Figure 6 and Figure 8, respectively). However, they are notably higher (by approximately 0.8 eV, for both PBE and HSE06) than the calculated bandgap energies for MA_2_ZrCl_6_ (Figure 4). Similarly, the calculated bandgap energy of (PH_4_)_2_ZrBr_6_ has values of 2.70 eV (PBE) and 3.79 eV (HSE06), which are close to those of (NH_4_)_2_ZrBr_6_, DMA_2_ZrBr_6_, and TMA_2_ZrBr_6_, but significantly lower than that of MA_2_ZrBr_6_. In the case of iodine anion, the band gap of (PH_4_)_2_ZrBr_6_ is found to be smaller, by ~0.4 eV, than that of (NH_4_)_2_ZrBr_6_ and very close (~0.1 eV larger) to that of TMA_2_ZrBr_6_, according to HSE06.

Upon halogen substitution, the band gap energies generally decrease. Specifically, replacing Cl with Br in (PH_4_)_2_ZrBr_6_ leads to a decrease of 0.8 eV (PBE) and 1.0 eV (HSE06). When Br is further replaced by I, a significant decrease in the bandgap energy, of 1.0 eV (PBE) and 1.2 eV (HSE06), is found.

Regarding the structural properties of the (PH_4_)_2_ZrX_6_ series (Table 5), it is observed that the increase in the molecular weight of the halogen significantly influences the lattice constants. Importantly, these compounds maintain their cubic crystal symmetry throughout the series. Specifically, for (PH_4_)_2_ZrCl_6_, the cubic lattice constant is *a*=10.34 Å. When chlorine (Cl^−^) is replaced by bromine (Br^−^), the lattice constant increases by 0.65 Å. Finally, upon substituting bromine (Br^−^) with iodine (I^−^), the lattice constant for (PH_4_)_2_ZrI_6_ increases further by 0.74 Å.

### 3.6. The Effect of A-Cation and X-Anion

**Figure 11:**
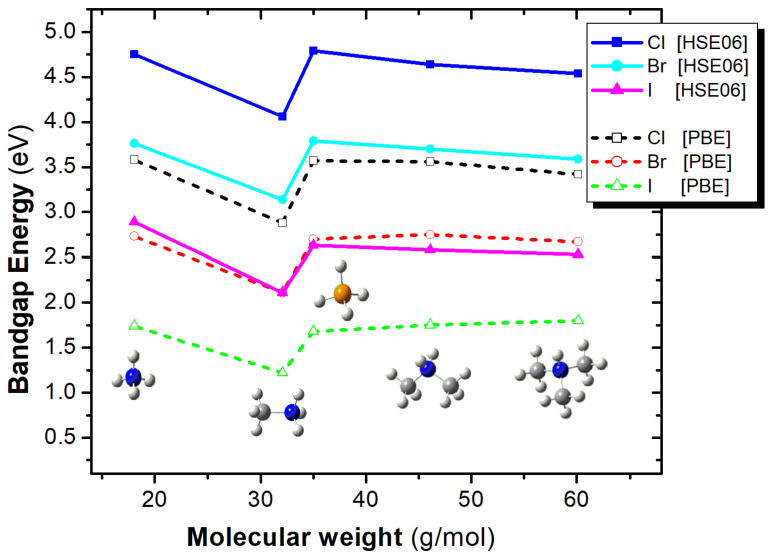
Bandgap energy as a function of the molecular weight of the A-site cation, for the A_2_ZrX_6_ crystal, calculated using the HSE06 (solid lines) and PBE (dash lines) functionals. Color assignment: blue for N, grey for carbon, white for hydrogen, yellow for S and orange for P.

Let us now shed more light on the effect of the A-site substitution and the X-site substitution on the bandgap energy. Regarding the A-site effect, for both functionals and for all X anions, MA in A-site results in the smallest bandgap energy, while the remaining four cations, i.e., NH_4_^+^, PH_4_^+^, DMA, and TMA, result in similar bandgap energies, as seen in Figure 11. Specifically, the bandgap energy has been plotted as a function of the molecular weight of the A-site cation, since molecular weight can be used as a proxy for the size of the molecular ion, which is responsible for the observed changes in the lattice parameters (Figure 11).

In order to explain the A-cation effect and specifically why MA present the smallest bandgap, the NH_4_^+^, MA, DMA, and TMA cations were energetically optimized using the B3LYP/6-31G(d,p) methodology [43,44] to calculate the atomic charges via the natural population analysis and the volume of these cations. It was found that the charges on the H atoms bonded on the N atoms are +0.50 e, while the N atom has a charge of about –1.0 e. The three H atoms of the MA attached to the N atom of MA have a similar charge of +0.49 e. On the contrary, the H atoms attached to N of the DMA and TMA have a charge of +0.37 e and +0.36 e, respectively. Thus, while all NH_4_^+^, MA, DMA, and TMA are positively charged by +1.0 e, the NH_4_^+^ and MA have the most positively charged H atoms that may affect the bandgap [45]. Comparing the NH_4_^+^ and MA cations, they have different volumes, i.e., about 4.7 Å and 5.7 Å. The second one fits better and a better size match at the A-site can reduce the band gap, though the relationship is not always linear and depends on other structural factors too [46]. As a result of the cation size and the charges of the H atoms attached to N, the systems with MA have the smallest band gap among the considered A-site cations.

The A-site cations’ shape and charge distribution also influence crystal symmetry. The NH_4_^+^ and PH_4_^+^ are both tetrahedral. The rest, especially DMA^+^ and TMA^+^, are elongated. Thus, it is not surprising that for NH_4_^+^ and PH_4_^+^ cations the optimal crystal structures are close to cubic. However, in the case of PH_4_^+^, the structure remains cubic, while for NH_4_^+^, it is, to a small extent, distorted towards triclinic symmetry. This difference can be attributed to the distribution of positive charge. Indeed, by performing the natural population analysis mentioned above, we found that, for NH_4_^+^, each hydrogen atom carries a positive charge of around +0.5 e, compared to only +0.1 e for PH_4_^+^. These tetrahedrally oriented charges, significantly larger for NH_4_^+^, lead to enhanced interactions with the octahedrally oriented halogen atoms and can explain the distortion of the cubic lattice towards triclinic. The interactions of PH_4_^+^ hydrogens with halogens are much weaker, and the structure prefers cubic symmetry. According to our previous work [35], compounds with trimethyl sulfonium cation (TMS^+^) at the A-site also adopt a cubic structure, which can also be attributed to the shape of the cation and the low positive charge (~ +0.2 e) on hydrogen atoms.

**Figure 12:**
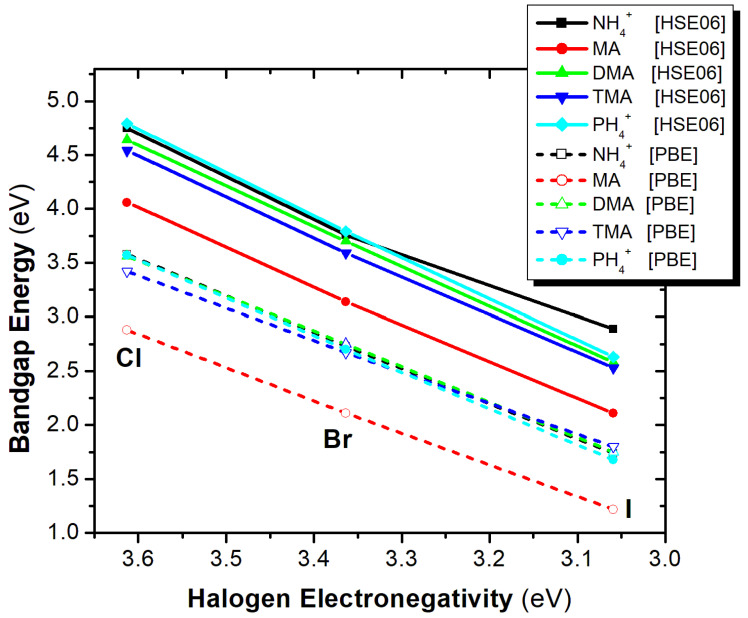
Bandgap energy as a function of halogen electronegativity of the A_2_ZrX_6_ compounds, calculated using the HSE06 (solid lines) and PBE (dash lines) functionals.

Regarding the effect of the X-site substitution, as the electronegativity of the X-site halogen is decreased, i.e., Cl > Br > I, the bandgap also decreases (see Figure 12). Both PBE and HSE06 functionals exhibit similar trends, with the smallest bandgap values observed for X = I. The observed linearity of these trends suggests a strong predictive capability, allowing for the reliable estimation of missing bandgap values for halogens in these or similar compounds.

## 4. Conclusions

In this density functional theory (DFT) study, we investigate the impact of A-site cation and X-site halogen substitutions on the structural and optoelectronic properties of lead-free A_2_ZrX_6_ “defect” perovskites. Following our previous work [35], we extend the A-site cation substitutions by considering four ammonium-derived cations (ammonium, methylammonium, dimethylammonium, trimethylammonium) as well as phosphonium. These cations are combined with three X-site halogens: Cl, Br, I. We demonstrate a significant tunability in the properties of these promising materials.

We show that both the size and composition of the A-site cation, as well as the electronegativity of the X-site anion, significantly influence the electronic band gap and lattice parameters. Our results offer a quantitative measure of this tunability. Specifically, we found that larger A-site cations lead to enlarged lattice constants. Furthermore, the bandgap energy is found to depend linearly on halogen electronegativity, with iodine-containing compounds systematically exhibiting, the lowest band gaps for all studied compounds. We find a broad range of predicted band gap values, spanning from wide bandgap materials (~4.79 eV for (PH_4_)_2_ZrCl_6_) suitable for UV detection or insulation, down to the lower end of the visible spectrum (~2.11 eV for MA_2_ZrI_6_), which holds promise for optoelectronic applications, like LEDs. It is worth mentioning that the (PH_4_)_2_ZrX_6_ series maintain cubic crystal symmetry across all halogens, in contrast to the triclinic symmetry found for the other ammonium-derived A-site cations.

The theoretical insights, presented in this work, demonstrate at a quantitative level, the vast potential of A-site cation and halogen substitution as powerful strategies for precisely tailoring the electronic and structural characteristics of A_2_ZrX_6_ “defect” perovskites. Finally, our calculations show that the MA cation yields the smallest band gap among the ones studied, a result that is attributed to its size and the charges of the hydrogen atoms attached to nitrogen. Our findings provide valuable theoretical predictions for future experimental synthesis and optimization efforts aimed at developing novel, high-performance, and lead-free perovskite materials for diverse optoelectronic applications.

## Figures and Tables

**Table 1:** Calculated bandgap energy, *E_gap_*, (in eV), using PBE and HSE06 functionals, crystal symmetry, the lattice constants (*a*, *b*, *c*) (in Å) and the lattice angles (*α*, *β*, *γ*) (in °) for the structures (NH_4_)_2_ZrX_6_.

				Lattice Constants			Lattice Angles		
Structure	*E_gap_* PBE	*E_gap_* HSE06	Symmetry of Crystal	*a*	*b*	*c*	*α*	*β*	*γ*
(NH_4_)_2_ZrCl_6_	3.58	4.75	triclinic	10.52	10.10	10.04	90.00	90.00	89.68
(NH_4_)_2_ZrBr_6_	2.73	3.76	triclinic	11.06	10.58	10.54	90.00	90.00	89.80
(NH_4_)_2_ZrI_6_	1.74	2.89	triclinic	11.87	11.35	11.34	90.00	90.00	90.04

**Table 2:** Calculated bandgap energy, *E_gap_*, (in eV), using PBE and HSE06 functionals, crystal symmetry, the lattice constants (*a*, *b*, *c*) (in Å) and the lattice angles (*α*, *β*, *γ*) (in °) for the structures MA_2_ZrX_6_.

				Lattice Constants			Lattice Angles		
Structure	*E_gap_* PBE	*E_gap_* HSE06	Symmetry of Crystal	*a*	*b*	*c*	*α*	*β*	*γ*
MA_2_ZrCl_6_	2.88	4.06	triclinic	11.26	10.84	11.18	90.00	90.00	89.98
MA_2_ZrBr_6_	2.11	3.14	triclinic	11.64	11.23	11.59	90.00	90.00	89.99
MA_2_ZrI_6_	1.22	2.11	triclinic	12.30	11.92	12.28	89.99	90.00	89.99

**Table 3:** Calculated bandgap energy, *E_gap_*, (in eV), using PBE and HSE06 functionals, crystal symmetry, the lattice constants (*a*, *b*, *c*) (in Å) and the lattice angles (*α*, *β*, *γ*) (in °) for the structures DMA_2_ZrX_6_.

				Lattice Constants			Lattice Angles		
Structure	*E_gap_* PBE	*E_gap_* HSE06	Symmetry of Crystal	*a*	*b*	*c*	*α*	*β*	*γ*
DMA_2_ZrCl_6_	3.56	4.64	triclinic	11.21	12.68	11.41	89.21	88.68	89.07
DMA_2_ZrBr_6_	2.75	3.70	triclinic	11.55	13.29	11.64	89.30	89.28	91.20
DMA_2_ZrI_6_	1.84	2.46	triclinic	12.20	13.84	12.30	89.30	89.47	92.14

**Table 4:** Calculated bandgap energy, *E_gap_*, (in eV), using PBE and HSE06 functionals, crystal symmetry, the lattice constants (*a*, *b*, *c*) (in Å) and the lattice angles (*α*, *β*, *γ*) (in °) for the structures TMA_2_ZrX_6_.

				Lattice Constants			Lattice Angles		
Structure	*E_gap_* PBE	*E_gap_* HSE06	Symmetry of Crystal	*a*	*b*	*c*	*α*	*β*	*γ*
TMA_2_ZrCl_6_	3.42	4.54	triclinic	11.64	13.21	12.04	91.07	98.32	90.12
TMA_2_ZrBr_6_	2.67	3.59	triclinic	12.07	13.63	12.42	91.08	97.98	90.64
TMA_2_ZrI_6_	1.80	2.53	triclinic	12.67	14.25	13.03	91.03	97.16	91.01

**Table 5:** Calculated bandgap energy, *E_gap_*, (in eV), using PBE and HSE06 functionals, crystal symmetry and the lattice constants (*a*, *b*, *c*) (in Å) for the structures (PH_4_)_2_ZrX_6_.

				Lattice Constants		
Structure	*E_gap_* PBE	*E_gap_* HSE06	Symmetry of Crystal	*a*	*b*	*c*
(PH_4_)_2_ZrCl_6_	3.57	4.79	cubic	10.34	10.34	1034
(PH_4_)_2_ZrBr_6_	2.70	3.79	cubic	10.99	10.99	10.99
(PH_4_)_2_ZrI_6_	1.68	2.63	cubic	11.73	11.73	11.73

## Data Availability

The original contributions presented in this study are included in the article. Further inquiries can be directed at the corresponding authors.
